# Sentinel Lymph Node Detection in Cervical Cancer: Challenges in Resource-Limited Settings with High Prevalence of Large Tumours

**DOI:** 10.3390/jcm14041381

**Published:** 2025-02-19

**Authors:** Szilárd Leó Kiss, Mihai Stanca, Dan Mihai Căpîlna, Tudor Emil Căpîlna, Maria Pop-Suciu, Botond Istvan Kiss, Szilárd Leó Kiss, Mihai Emil Căpîlna

**Affiliations:** 1 First Obstetrics and Gynaecology Clinic, George Emil Palade University of Medicine, Pharmacy, Science, and Technology of Targu Mures, 540142 Targu Mures, Romania; doctorstanca@gmail.com (M.S.); kszilardl@yahoo.com (S.L.K.S.); mcapilna@gmail.com (M.E.C.); 2 Faculty of General Medicine, George Emil Palade University of Medicine, Pharmacy, Science, and Technology of Targu Mures, 540142 Targu Mures, Romania; capilnadan@gmail.com (D.M.C.); dodocapilna@yahoo.com (T.E.C.); 3 Department of Nuclear Medicine, Emergency County Hospital Targu Mures, 540136 Targu Mures, Romania; mereuta_maria@yahoo.com; 4 Second Surgery Clinic, George Emil Palade University of Medicine, Pharmacy, Science, and Technology of Targu Mures, 540142 Targu Mures, Romania; drkissbotondi@gmail.com

**Keywords:** sentinel lymph node mapping, cervical cancer, lymphatic metastases, resource-limited settings, radiotracer, parametrial lymph nodes

## Abstract

**Background/Objectives**: Cervical cancer primarily disseminates through the lymphatic system, with the metastatic involvement of pelvic and para-aortic lymph nodes significantly impacting prognosis and treatment decisions. Sentinel lymph node (SLN) mapping is critical in guiding surgical management. However, resource-limited settings often lack advanced detection tools like indocyanine green (ICG). This study evaluated the feasibility and effectiveness of SLN biopsy using alternative techniques in a high-risk population with a high prevalence of large tumours. **Methods**: This prospective, observational study included 42 patients with FIGO 2018 stage IA1–IIA1 cervical cancer treated between November 2019 and April 2024. SLN mapping was performed using methylene blue alone or combined with a technetium-99m radiotracer. Detection rates, sensitivity, and false-negative rates were analysed. Additional endpoints included tracer technique comparisons, SLN localization patterns, and factors influencing detection success. **Results**: SLNs were identified in 78.6% of cases, with bilateral detection in 57.1%. The combined technique yielded higher detection rates (93.3% overall, 80% bilateral) compared to methylene blue alone (70.4% overall, 40.7% bilateral, *p* < 0.05). The sensitivity and negative predictive values were 70% and 93.87%, respectively. Larger tumours (>4 cm), deep stromal invasion, and prior conization negatively impacted detection rates. False-negative SLNs were associated with larger tumours and positive lymphovascular space invasion. **Conclusions**: SLN biopsy is feasible in resource-limited settings, with improved detection rates using combined tracer techniques. However, sensitivity remains suboptimal due to a steep learning curve and challenges in high-risk patients. Until a high detection accuracy is achieved, SLN mapping should complement, rather than replace, pelvic lymphadenectomy in high-risk cases.

## 1. Introduction

The primary route of dissemination for cervical cancer is through the lymphatic system, affecting the pelvic and para-aortic lymph nodes. The incidence of lymphatic metastases increases with disease stage. According to Olthof, the risk of pelvic lymph node metastases is 2% in stage IA2, 14–36% in stage IB, 38–51% in stage IIA, and 47% in stage IIB [1]. Lymphatic metastases in cervical cancer tend to spread sequentially, first draining to the pelvic lymph nodes, followed by the para-aortic nodes [2]

The presence of lymphatic metastases is an important prognostic factor, as survival is inversely proportional to the number of lymph nodes positive for metastasis [3]. For these reasons, the 2018 FIGO staging system incorporated the presence of lymph node metastases [4].

The identification of metastatic lymph nodes is pivotal in determining the optimal treatment strategy; therefore, lymph node assessment should be the initial step in the surgical management of cervical cancer. According to the European guidelines in stages IB1, IB2, and IIA1, without imaging-detected lymphadenopathy, the treatment of choice is surgical. If lymphatic metastases are identified, surgical treatment should be abandoned, and definitive chemo-radiotherapy is recommended. Sentinel lymph node (SLN) biopsy should be performed prior to pelvic lymphadenectomy. Indocyanine green (ICG) is the preferred technique for SLN mapping, while a combination of blue dye and radiocolloid serves as an alternative approach [5].

Romania has the highest incidence of cervical cancer in Europe, with 3368 new cases reported in 2022 [6]. Unfortunately, due to an inadequate screening programme, many cases are diagnosed in advanced stages. In our centre, patients admitted for surgery with early-stage cervical cancer often present with tumours larger than 2 cm, along with additional risk factors such as deep stromal invasion and lymphovascular space invasion (LVSI) [7].

The aim of our study was to evaluate the applicability of the SLN technique in a resource-limited setting where ICG is not available, with a high incidence of intermediate and high-risk tumours. The primary endpoints were to determine the detection rate of SLNs and assess the sensitivity of SLN biopsy for identifying lymph node metastases. Secondary endpoints included comparing the effectiveness of different tracer techniques, identifying the number and localization of SLNs, analysing factors that influence the detection rate, and evaluating the involvement of parametrial lymph nodes.

## 2. Materials and Methods

### 2.1. Study Design and Population

This prospective, observational study was conducted at the 1st Obstetrics and Gynaecology Clinic of the Emergency Clinical County Hospital in Târgu Mureș, affiliated with the George Emil Palade University of Medicine, Pharmacy, Science, and Technology of Târgu Mureș, between November 2019 and April 2024. Ethical approval was obtained from the institutional ethics committee (no.33680/13 December 2019), and all participants provided written informed consent before enrolment. The study cohort comprised women diagnosed with histopathologically confirmed cervical cancer, classified as FIGO 2018 stages IA1 with LVSI, IA2, IB1 to IB3, and IIA1, with no clinical or radiological evidence of lymph node involvement. The preoperative evaluation of operability included imaging studies (CT or MRI) and clinical gynaecological examinations performed by specialists in gynaecological oncology and cancer surgery.

### 2.2. Surgical Treatment

Patients were assigned to different surgical treatments based on their clinical stage, previous interventions, and the desire to preserve fertility. Low-risk patients (Stage IA1, IA2) underwent a radical hysterectomy type B2 according to the Querleu–Morrow classification. Patients post-conization with no residual macroscopic tumour underwent a laparoscopic radical hysterectomy type C2. Patients with stage IB1 to IB3, IIA1 underwent a radical hysterectomy type C2 via laparotomy. Four patients underwent a fertility sparing procedure with radical trachelectomy type C2. In all cases, SLN identification was performed, followed by pelvic lymphadenectomy.

### 2.3. SLN Identification

SLN identification was performed using methyl blue dye alone or in combination with a radiotracer. When a radiotracer was used, preoperative lymphoscintigraphy was conducted either on the day of surgery or the day before in the Nuclear Medicine Department. The lymphoscintigraphy procedure was executed according to a protocol adapted to the guidelines established by the European Association of Nuclear Medicine, as well as national standards. The procedure comprised several phases: initially, the injectable administration of a radiotracer was performed, followed by the acquisition of preoperative images, the intraoperative localization of the lesions identified through imaging utilising a gamma probe, and concluding with the excision of the identified lymph nodes.

Peritumoral/periorificial injections were administered in the four quadrants of the cervix using a 20 or 22 G needle. Injections into the necrotic areas of larger tumours were avoided.

Doses of approximately 110 MBq in a total volume of 2 mL (0.5 mL per deposit) of 99mTc-nanocolloidal albumin (Nano-HAS ROTOP Pharmaka GmbH, Dresden, Germany or NanoScan Medi-Radiopharma Ltd., Érd, Hungary) were utilised, adjusting the administered activity accordingly. Image acquisitions were conducted post-injection with dynamic imaging immediately following the injection (10 min, 1 min/frame), as well as segmental static images (3–5 min, early and late) from anterior, posterior, and lateral perspectives at 15 min and 60 min post-injection, respectively. The early acquisitions highlighted lymphatic ducts and the initial drainage lymph nodes, while the late acquisitions differentiated the SLNs from nodes representing other stations along the lymphatic route. Finally, SPECT acquisition was performed immediately after the late image acquisitions, a technique providing enhanced contrast and spatial resolution, along with a three-dimensional reference, which was particularly advantageous for detecting parametric or atypical locations.

### 2.4. Intraoperative SLN Identification

Immediately before laparotomy and after the induction of general anaesthesia, 4 mL of methyl blue dye was injected into the cervix at the 3 and 9 o’clock positions using a 26G needle, with 2 mL administered on each side at a depth of approximately 1 cm. Intraoperative SLN identification was conducted using the Europrobe 3.2 console (Eurorad, S.A. Eckbolsheim, France) for radiotracer detection. After the development of retroperitoneal spaces, lymph node regions were examined with the gamma probe to detect radioactivity and blue-stained lymphatic channels and nodes were visually inspected. Lymph nodes with significantly higher activity than the surrounding tissue, or those stained blue, were considered SLNs. If multiple lymph nodes were identified, the one with the highest radioactivity or the proximal, blue-stained node was designated as the SLN. Radio-labelled nodes were also verified ex vivo.

Seven anatomical regions were identified as described by Cibula et al.: the external iliac (right and left), the interiliac or obturator (right and left), the common iliac (right and left), and the presacral region [8]. The localisation of the SLNs was noted according to these regions. If SLNs were identified in multiple regions, they were submitted for pathology analysis separately.

### 2.5. Histopathological Analysis of the SLNs

All SLNs were sent separately for histopathological examination. Pathological review was performed by pathologists specialised in gynaecologic oncology. SLNs were processed according to an ultrastaging protocol, wherein each SLN was sectioned every 2 mm. Each fragment was embedded in a separate paraffin block and cut with a microtome into 4-micron sections. The block was sectioned until depletion to ensure thorough examination.

## 3. Results

A total of 42 patients were enrolled in the study, with a mean age of 48 years (range: 22–71). Of these, 19 patients (45.2%) were premenopausal, while 23 (54.8%) were postmenopausal. The most common histological subtype was squamous cell carcinoma, observed in 35 patients (83.3%), followed by adenocarcinoma in 4 cases (9.5%) and adenosquamous carcinoma in 3 cases (7.1%).

Regarding preoperative tumour stages, small tumours were rare, with stage IA1 being identified in one case (2.4%) and stage IA2 in two cases (4.8%). Intermediate tumours were the most frequent, comprising 12 cases (28.6%) of stage IB1 and 18 cases (42.9%) of stage IB2. Large tumours were observed in seven cases (16.7%) of stage IB3 and two cases (4.8%) of stage IIA1. Seven patients had undergone conization prior to their inclusion in the study.

Radical hysterectomy type C2 was performed in 33 cases (78.6%), laparoscopic radical hysterectomy type C2 in 3 cases (7.1%), abdominal radical type C2 trachelectomy in 4 cases (9.5%), and radical hysterectomy type B2 in 2 cases (4.8%). Patient characteristics are presented in Table 1.

According to the postoperative histopathological analysis, 10 cases (23.8%) had tumours smaller than 2 cm, 23 cases (54.8%) had tumours measuring between 2 and 4 cm, and 9 cases (21.4%) had tumours larger than 4 cm. The depth of invasion was less than one-third of the cervical stroma in 1 case (2.4%), between one-third and two-thirds in 6 cases (14.3%), and greater than two-thirds in 25 cases (59.5%). Parametrial involvement was observed in five patients (11.9%). LVSI was positive in 15 patients (35.7%) and negative in 25 patients (59.5%). According to the Sedlis criteria, 17 patients (40.5%) were classified as low risk, 10 patients (23.8%) as intermediate risk, and 15 patients (35.7%) as high risk.

The mean number of pelvic lymph nodes retrieved per side was 20.84. Parametrial lymph nodes were observed in 32 patients (76%). Metastatic lymph nodes were found in 14 patients (33.3%), with metastases detected in the parametrial lymph nodes in 8 cases (19%). In two cases (4.8%), the parametrial lymph node was the sole site of lymphatic metastasis without direct spread to the parametria. Pelvic nodal metastasis was associated with positive parametrial lymph nodes in 57% of cases. The histopathological characteristics are presented in Table 2.

**Figure 1:**
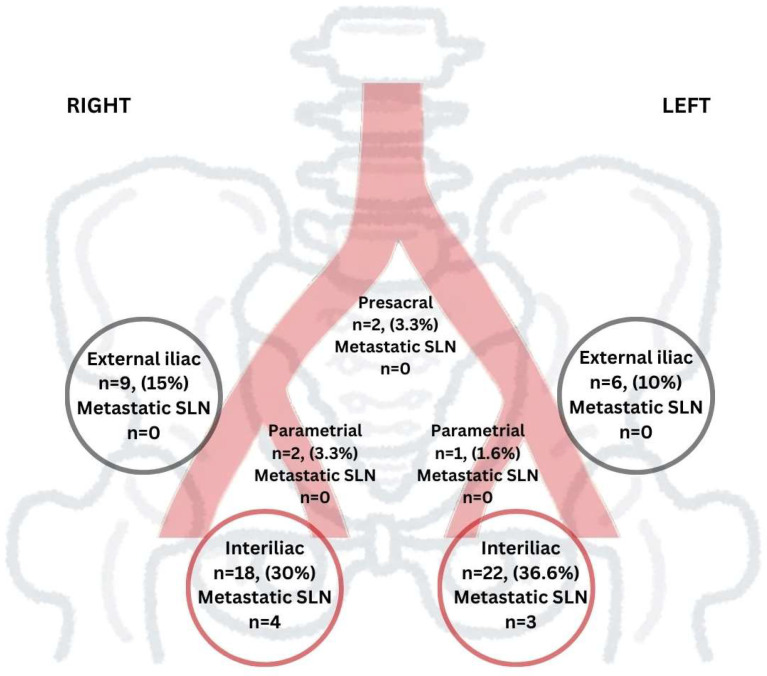
Localization of the SLNs.

SLNs were detected in 33 cases (78.6%), with a mean of 1.70 SLNs retrieved per side (range: 1–10). The localisation of the SLNs can be observed in Figure 1. The atypical localization of the SLN was observed in five cases (15%): presacral in two cases and parametrial in three cases.

In two cases, SLNs were detected along two different lymphatic routes within the same hemipelvis: in one case, bilaterally in the external iliac and obturator regions, and in the other case, in the interiliac region and parametrium on the right side.

SLN detection was achieved using two techniques: a combined approach with technetium and methyl blue in 15 cases (36%) and methyl blue alone in 27 cases (64%). Overall, at least one SLN was detected in 78.6% of cases, with bilateral detection achieved in 57.1% of cases.

Detection rates, as presented in Table 3, were higher with the combined blue dye and radiotracer technique (93.3% overall, 80.0% bilateral) compared to blue dye alone (70.4% overall, 40.4% bilateral, (*p* = 0.123) and (*p* = 0.014)), with a statistically significant improvement in the bilateral detection. Interestingly, detection was higher in larger tumours (>2 cm: 81.3% overall, 56.3% bilateral) compared to smaller ones (<2 cm: 70.0% overall, 50.0% bilateral, *p* = 0.660 and *p* = 1.000). On the other hand, SLN detection rates in patients with tumours < 4 cm were higher (81.8% overall, 63.6% bilateral) compared to those with tumours > 4 cm (66.7% overall, 22.2% bilateral). While the difference in the overall detection rates was not statistically significant (*p* = 0.375), the difference in the bilateral detection rates approached significance (*p* = 0.055). Additionally, cases with less stromal invasion (<2/3: 100% overall, 71.1% bilateral) showed better rates than those with greater invasion (>2/3: 80.0% overall, 60.0% bilateral, *p* = 0.560 and *p* = 0.683).

Patients with negative LVSI had higher detection rates (86.7% overall, 60.0% bilateral) compared to those with positive LVSI (72.0% overall, 48.0% bilateral, (*p* = 0.440) and (*p* = 0.462)). Younger patients (<45 years) had superior detection rates (88.2% overall, 76.5% bilateral) compared to older patients (>45 years: 72.0% overall, 40.0% bilateral, (*p* = 0.271) and (*p* = 0.020)), with a significant difference observed for bilateral detection. Patients after conization had slightly lower detection rates (71.0% overall, 42.9% bilateral) compared to non-conization cases (80.0% overall, 57.1% bilateral, (*p* = 0.631) and (*p* = 0.682)). Most differences were not statistically significant, except for the improvement in bilateral detection with the combined technique and among younger patients with tumours < 4 cm.

**Figure 2:**
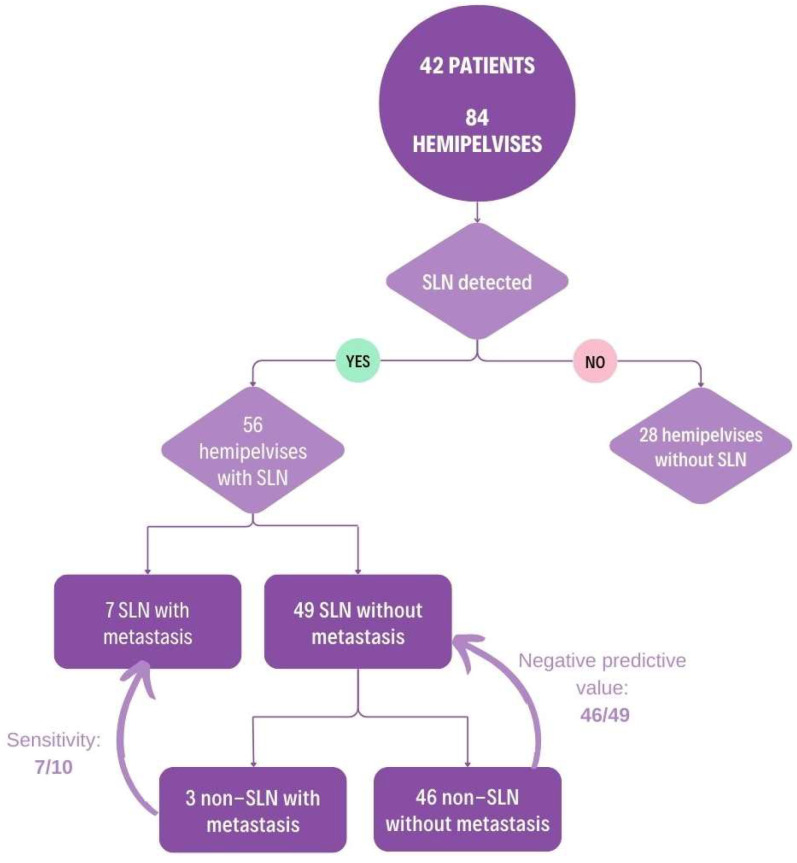
Sensitivity and negative predictive value.

Sensitivity and negative predictive values were calculated as presented in Figure 2.

From a total of eighty-four hemipelvises, in fifty-six, we identified at least one SLN. In seven hemipelvises, the SLNs were positive: four on the right side and three on the left side. Six nodes presented macrometastasis, and one node presented micrometastasis. In the 49 hemipelves with negative SLNs, we found 3 cases where non-SLNs were positive: 2 on the right side and 1 on the left side. The sensitivity of SLN detection was calculated as 70% (7/10). The negative predictive value was 93.87% (46/49), while the false-negative rate was 30% (3/10).

Among the 3 cases with false-negative SLNs, the tumour size was greater than 4 cm in all cases and LVSI was positive in each case. Stromal invasion exceeded 75% in all instances. Parametrial involvement was detected in one case. In terms of detection technique, two cases used methylene blue alone, while one case used a combination of methylene blue and Tc-99. Bilateral SLN detection was achieved in only one of these cases.

## 4. Discussion

Our study aimed to investigate whether SLN detection can reliably determine the presence of lymph node metastases in patients with a high prevalence of tumours larger than 2 cm in a resource-limited setting where advanced detection methods, such as the use of ICG, are unavailable. Among the detection methods employed, methylene blue dye was the most frequently used (64%).

The detection rate we achieved (93.3% overall, 80.0% bilateral) with radiocolloid utilisation approached the excellent results reported in several studies [9,10]. However, when methylene blue was used alone, our detection rates were considerably lower (70.4% overall, 40.7% bilateral). Staining techniques have been extensively studied, with the ICG technique being regarded as the most accurate [11,12,13]. However, some studies suggest that the choice of tracer does not significantly impact the success of the method [8,14]. In our study, a statistically significant difference was observed in the bilateral detection rate between the two methods.

While our detection rates with radiocolloid and blue dye were satisfactory, achieving an 80% bilateral detection rate, they remain lower than those reported with ICG in most studies [9,11,12,13,14,15,16,17]. To better illustrate this, we compared our findings with previously published studies in Table 4.

This aligns with the findings of Di Martino et al., who reported particularly high bilateral detection rates, especially in tumours greater than 2 cm. Given that our cohort also included a substantial proportion of large tumours, it is likely that the use of ICG could have enhanced our detection rates and overall sensitivity. Additionally, recent meta-analyses have consistently demonstrated that ICG achieves the highest bilateral detection rates, reinforcing its superiority in sentinel lymph node mapping [18,19,20].

In our study, we observed higher detection rates in larger tumours (>2 cm: 81.3% overall, 56.3% bilateral) compared to smaller tumours (<2 cm: 70.0% overall, 50.0% bilateral, *p* = 0.660 and *p* = 1.000), though these differences were not statistically significant. This finding contrasts with previous studies that have reported lower detection rates or higher false-negative rates in larger tumours. For instance, Tanaka et al. found that patients with tumour diameters ≥ 2 cm had a significantly higher false-negative rate compared to those with smaller tumours (8.6% vs. 1.4%, *p* < 0.01) [21]. Similarly, Altgassen et al. reported a reduced negative predictive value in larger tumours (88.5% vs. 99.1%, *p* < 0.01) [22]. The lack of statistical significance and the reverse trend in our study may be attributed to the small sample size of patients with smaller tumours (<2 cm, n = 10), which limits the power to detect significant differences. Additionally, prior conization, performed in 7 out of these 10 patients, might have influenced lymphatic drainage patterns, potentially reducing the detection rate in this subgroup. Patients with prior conization in our cohort had slightly lower detection rates (71.0% overall, 42.9% bilateral) compared to those without conization (80.0% overall, 57.1% bilateral, *p* = 0.631 and *p* = 0.682), though the differences were not statistically significant. A small study by Dargent previously reported a significant reduction in the detection rates following conization. [23] However, more recent studies have shown no statistically significant reduction, aligning with our findings [14,15]. This suggests that while conization may impact detection rates in some cases, its effect is likely variable and not consistently significant across larger cohorts.

According to Cibula et al., a lower detection rate and the omission of tumour-positive SLNs may be attributed to challenges in the dye application to the residual cervical stroma. Large tumours with central necrosis can lead to retrograde dye leakage into the vagina or inaccurate dye placement in the parametria. Proper technique is crucial, requiring the preoperative identification of the residual stroma, use of a long spinal needle, and prevention of dye escape during application [24]. Dostalek et al. demonstrated that with the proper injection technique and careful application of the tracer into the residual tumour-free cervical stroma, similar bilateral detection rates (79%, 83%, and 76% for tumours > 2 cm, 2–3.9 cm, and ≥4 cm, respectively) and sensitivity (93.3%, 93.3%, and 100% for tumours > 2 cm, 2–3.9 cm, and ≥4 cm, respectively) can be achieved regardless of tumour size [17]. In our series, deep stromal invasion was a common finding, observed in 59.5% of the patients. This high prevalence likely impacted the technical aspects of tracer injection into the healthy cervical stroma, which was often challenging in these cases and may have compromised the success rate of SLN detection. This is reflected in our results, as cases with less stromal invasion (<2/3) showed better detection rates (100% overall, 71.1% bilateral) compared to those with greater invasion (>2/3: 80.0% overall, 60.0% bilateral), although the differences were not statistically significant.

In our patient cohort, SLN analysis confirmed lymphatic metastases in 70% of cases. While some studies have reported similar results [22], validation studies of the method describe sensitivities approaching 100% [8,9,14,25,26]. However, it should be noted that most of these studies were performed in specialised centres with extensive expertise in SLN detection, where the use of more efficient tracers was more common.

One possible explanation for our suboptimal results is that we are at the early stages of the learning curve, as SLN mapping was implemented at our institution simultaneously with the initiation of this study. This is consistent with findings from other studies; for instance, Kim et al. reported that a surgical learning period of at least 27 cases is required for effective SLN mapping in gynaecologic cancer [27].

Our high rate of false-negative results can indicate that in most cases, we successfully identified one SLN but missed the one containing the metastasis. This finding raises the question of the potential existence of multiple lymphatic drainage pathways from the cervix. Ercoli et al., through cadaveric dissection, identified three distinct lymphatic pathways for cervical lymph drainage based on the level where lymph channels exit the utero-vaginal fascia: the supraureteral paracervical pathway, the infraureteral paracervical pathway, and the neural paracervical pathway. The supraureteral paracervical pathway was observed most frequently, serving as the sole drainage route in only 70% of cases. This supports the notion that lymphatic vessels from the cervix may drain to multiple SLNs within the pelvis [28]. Geppert et al., analysing the localization of SLNs in endometrial cancer, described two consistent pelvic lymphatic pathways: an upper paracervical pathway (UPP), draining to external and/or obturator nodes, and a lower paracervical pathway (LPP) draining to internal iliac and/or presacral nodes. The bilateral display of at least one pathway occurred in 98%, while both pathways were bilaterally displayed in 30% of cases. The LPP was less frequently observed but harboured metastases in nearly one-third of node-positive patients [29]. These studies support the concept of multiple lymphatic drainage pathways from the cervix and the possibility of more than one SLN in each hemipelvis. In our series, multiple lymphatic pathways were identified in two cases: in one case, SLNs were located bilaterally in the external iliac and obturator regions, while in the other, SLNs were found in the interiliac region and the parametrium on the right side.

In our cohort, patients had a higher rate of parametrial lymph node presence (76%) and a higher rate of parametrial node metastasis (19%) compared to other recent studies [10,30,31,32]. In two cases (4.8%), the parametrial lymph node was the sole site of lymphatic metastasis, occurring without direct tumour spread to the parametrial tissue. This finding raises the question of whether these patients should be staged as IIB or IIIC, as suggested by other authors [10,30]. It also highlights the importance of the proper removal of paracervical tissue to avoid understaging and to ensure that these patients receive appropriate adjuvant treatment [31].

A key strength of this prospective observational study is its focus on a distinctive patient cohort with a high prevalence of large cervical tumours featuring deep stromal invasion, managed in a resource-limited setting where the most commonly used tracer is blue dye. Moreover, the elevated proportion of node-positive cases offers valuable insight into SLN detection in more advanced disease.

However, several limitations must be acknowledged. The unavailability of more efficient tracer technologies, such as ICG, may reduce the accuracy of SLN mapping, while the relatively small sample size constrains the study’s statistical power. Limited institutional experience in SLN detection further contributes to the reduced diagnostic precision observed in this setting.

This study aims to offer an example for clinicians facing similar circumstances, illustrating the possible outcomes in a resource-limited environment with a high prevalence of high-risk tumours, as well as the specific limitations of the SLN method arising in such situations.

## 5. Conclusions

In this study, we demonstrate the feasibility of SLN biopsy in a resource-limited setting with high-risk cervical cancer. The combined use of technetium and methylene blue improved detection rates, although overall sensitivity was suboptimal, reflecting a steep learning curve. Larger tumours, deep stromal invasion, and conization posed challenges, while frequent parametrial lymph node metastases emphasised thorough surgical assessment. In this setting (high-risk tumours, no ICG), relying solely on the SLN technique without complete lymphadenectomy would lead to high false-negative results. SLN-only protocols may become viable once proficiency is achieved and enhanced detection rates and sensitivity are attained.

## Figures and Tables

**Table 1:** Patient characteristics.

**Number of patients**	42
**Age median, years (range)**	48 years (22–71)
**Menopausal status:**	
Premenopausal	19 (45.2%)
Postmenopausal	23 (54.8%)
**No. patients by preoperative FIGO 2018 staging:**	
IA1	1 (2.4%)
IA2	2 (4.8%)
IB1	12 (28.6%)
IB2	18 (42.9%)
IB3	7 (16.7%)
IIA1	2 (4.8%)
**Type of surgery:**	
Radical Hysterectomy Type C2	33 (78.6%)
Laparoscopic Radical Hysterectomy Type C2	3 (7.1%)
Abdominal Radical Type C2 Trachelectomy	4 (9.5%)
Radical Hysterectomy Type B2	2 (4.8%)
**Prior conization**	7 (16.7%)

**Table 2:** Histopathological characteristics.

**Tumour histology:**	
Squamous cell carcinoma, total	35 (83.3%)
Adenocarcinoma	3 (7.1%)
Adenoscuamous carcinoma	4 (9.5%)
**Tumour size:**	
Mean tumour size mm (range)	33.03 (3–80)
No. of cases with tumour size	
<2 cm	10 (23.81%)
2–4 cm	23 (54.76%)
>4 cm	9 (21.43%)
**Depth of invasion**	
<1/3	1 (2.4%)
1/3–2/3	6 (14.3%)
>2/3	25 (59.5%)
**LVSI**	
Absent	15 (35.7%)
Present	25 (59.5%)
**Direct parametrial involvement**	5 (11.9%)
**Lymph node metastases**	14 (33.3%)
**Nr. of pelvic lymph nodes retrieved:** mean (range)	20.84 (4–56)
**Parametrial lymph nodes:**	
Nr. of patients with parametrial lymph nodes	32 (76%)
Metastatic parametrial lymph nodes	8 (19%)
Metastasis only in parametrial lymph nodes	2 (4.8%)
**Nr. of SLNs retrieved per side:** mean (range)	1.70 (1–10)
**Nr. of hemipelvises with metastatic SLN:**	7 (12.5%) ^1^
Macrometastasis	6 (10.71%) ^1^
Micrometastasis	1 (1.79%) ^1^

^1^ Based on 56 hemipelvises where SLN was detected.

**Table 3:** SLN detection.

	Overall Detection		Bilateral Detection	
**SLN detection technique** (n)		*p* ^1^		*p* ^1^
Total (42)	78.6%		57.1%	
Blue dye only (27)	70.4%	0.123	40.4%	0.014
Blue dye + radiotracer (15)	93.3%		80.0%	
**Tumour size** (n)				
<2 cm (10)	70.0%	0.660	50.0%	1.000
>2 cm (32)	81.3%		56.3%	
<4 cm (26)	81.8%	0.375	63.6%	0.055
>4 cm (9)	66.7%		22.2%	
**Stromal invasion** (n)				
<2/3 (7)	100%	0.560	71.1%	0.683
>2/3 (25)	80.0%		60.0%	
**LVSI** (n)				
Present (25)	72.0%	0.440	48.0%	0.462
Absent (15)	86.7%		60.0%	
**Age** (n)				
<45 (25)	88.2%	0.271	76.5%	0.020
>45 (17)	72.0%		40.0%	
**Conization**				
Yes (7)	71%	0.631	42.9%	0.682
No (35)	80%		57.1%	

^1^ The chi-square test or Fisher’s exact test was used, as appropriate, based on the distribution and size of the data. A *p*-value of less than 0.05 was considered statistically significant.

**Table 4:** Comparison of tracers.

	Number of Patients	Stage (FIGO 2009)	Nr. of Tumours > 2 cm (%)	Bilateral SLN Detection Rate		Sensitivity
				Tc99 + Blue Dye	ICG	
Our study	42	IA1-IIA2	32 (76.2%)	80%	-	70%
Imboden et al. (2015) [13]	58	IA1-IIB	39 (67%)	61%	95.5	100%
Buda et al. (2016) [16]	144	IA1-IB1	65 (45%)	76.3%	98.5%	96%
Salvo et al. (2017) [14]	188	IA1-IIA1	55 (29%)	67%	57%	96.4%
Di Martino et al. (2017) [12]	95	IB1-IIB	95 (100%)	66%	91.7%	88.5%
Buda et al. (2018) [15]	65	IA1-IB2	NI	69.6%	95.2%	100%
Dostalek et al. (2018) [17]	350	IA-II	210 (60%)	80.3%	-	96%
O. Lührs et al. (2020) [11]	65	IA1-IIA	NI	60%	98.5%	100%
Sponholtz et al. (2021) [9]	245	IA1-IB2	103 (42%)	-	82% (80.9% *)	96.3% *

* Calculated only for tumours larger than 2 cm.

## Data Availability

The data presented in this study are available on request from the corresponding author.
